# Cyclin-dependent kinase 4/6 inhibitors combined with stereotactic ablative radiotherapy in oligometastatic HR-positive/HER2-negative breast cancer patients

**DOI:** 10.1093/bjr/tqae138

**Published:** 2024-08-09

**Authors:** Marcin Kubeczko, Dorota Gabryś, Aleksandra Krzywon, Michał Jarząb

**Affiliations:** Breast Cancer Center, Maria Sklodowska-Curie National Research Institute of Oncology Gliwice Branch, 44-102 Gliwice, Poland; Department of Radiotherapy, Maria Sklodowska-Curie National Research Institute of Oncology Gliwice Branch, 44-102 Gliwice, Poland; Department of Biostatistics and Bioinformatics, Maria Sklodowska-Curie National Research Institute of Oncology Gliwice Branch, 44-102 Gliwice, Poland; Breast Cancer Center, Maria Sklodowska-Curie National Research Institute of Oncology Gliwice Branch, 44-102 Gliwice, Poland

**Keywords:** cyclin-dependent kinase 4/6 inhibitors, stereotactic ablative radiotherapy, oligometastatic disease

## Abstract

**Objectives:**

Cyclin-dependent kinase 4/6 inhibitors (CDK4/6i) have significantly improved the survival of patients with hormone receptor-positive HER2-negative advanced breast cancer (ABC). Although stereotactic ablative radiotherapy (SABR) is used more often in routine clinical practice, data on the safety and efficacy of combining SABR with CDK4/6i are lacking. Herein, we present the results of SABR combined with CDK4/6i in ABC.

**Methods:**

Patients with ABC who received CDK4/6i and SABR between 2018 and 2023 were analysed.

**Results:**

Among 384 patients treated with CDK4/6i, 34 patients received 44 courses of SABR. Two-year progression-free survival (PFS) was 63.6% (95% CI, 45.8-88.3), and the median PFS was 32 months. Three-year overall survival (OS) was 88.9% (95% CI, 77.7-100). Two-year local control (LC) was 92.7% (95% CI, 83.4-100). Median OS and LC were not reached. The subgroup analysis showed the difference in survival between oligometastatic patients (OMD) and non-OMD subgroup. Two-year PFS was 69.2% (95% CI, 44.5-100) in OMD compared with 57.4% (95% CI, 36-91.7) in the non-OMD (*P* = .042). Three-year OS was 90% (95% CI, 73.2-100) in OMD compared with 86.2% (95% CI, 70-100) in the non-OMD (*P* = .67). Median PFS and OS in the non-OMD were 26 and 56 months, respectively, and were not reached in OMD. Fifteen patients required CDK4/6i dose reduction, and 2 discontinued treatment due to toxicity. No difference in high-grade toxicity was observed between the sequential and concurrent SABR.

**Conclusion:**

The addition of SABR to CDK4/6i seems to be safe and effective, especially in patients with oligometastatic disease.

**Advances in knowledge:**

In advanced breast cancer patients treated with CDK4/6i, SABR provides a high local control and may provide additional benefit in an oligometastatic setting.

## Introduction

Cyclin-dependent kinase 4/6 inhibitors (CDK4/6i) have significantly improved the survival of patients with hormone receptor-positive (HR+) human epidermal growth factor receptor 2-negative (HER2−) advanced breast cancer (ABC).^1^ To date, 3 CDK4/6i have been approved by the US Food and Drug Administration and European Medicines Agency for the first-line and second-line treatment of ABC. Furthermore, abemaciclib was approved for the adjuvant treatment of high-risk early breast cancer.^2^ For patients with HR+/HER2− ABC, current National Comprehensive Cancer Network and European Society for Medical Oncology guidelines recommend CDK4/6i in combination with endocrine therapy as the preferred first-line treatment.^3^^,^^4^ Despite improvements in systemic treatment, many breast cancer patients require radiation therapy in the palliative setting due to symptomatic disease.^5^

External beam radiation therapy (EBRT) for bone metastasis is one of the most frequent utilization of palliative radiotherapy (RT), providing efficient pain relief.^6^ Stereotactic body RT (SABR) delivers targeted radiation to the tumour while minimizing radiation to adjacent normal tissue. It allows the therapy of tumours in a single fraction or a limited number of fractions. In the therapy of painful bone metastases, SABR is used not only as a primary therapy but also as retreatment of recurrent or persistent bone pain after EBRT.^7^ Moreover, in specific patients’ subsets, SBRT might be preferred over EBRT, for example, definitive treatment of bone metastases in patients with relatively radioresistant histologies like sarcoma.^8^

Patients with oligometastatic bone metastases might also be candidates for SABR, mainly when the primary site is controlled and estimated survival exceeds 6 months. The oligometastatic disease is usually defined as a low-volume metastatic disease with a limited number and size of metastatic lesions.^9^ Uncertainty remains concerning the exact meaning of limited metastases; however, trial design and clinical practice today are relatively consistent in limiting oligometastatic disease to a maximum of 5 metastases and 3 organs.^10^ Importantly, each lesion should potentially qualify for local therapy to achieve complete remission.

The Stereotactic Ablative Radiotherapy for the Comprehensive Treatment of Oligometastases trial (SABR-COMET) was a multicentre, open-label, phase 2 randomized study.^11^ Patients with multiple cancer types were included, also with breast cancer, a common approach in trials of metastasis-directed stereotactic RT. Addition of SABR to the palliative standard of care results in prolonged overall survival (OS); however, the difference was not statistically significant.

Although SABR is used more often in routine clinical practice, there is a paucity of data on the safety and efficacy of combining SABR with targeted therapy. Recently, consensus recommendations on metastases-directed stereotactic body RT in combination with targeted therapy or immunotherapy were published by the EORTC-ESTRO OligoCare consortium.^12^ Due to limited retrospective data on the combination of CDK/6i and SABR, no consensus was reached on whether or not CDK4/6 inhibitors can be administered on the same days as SABR delivery. Furthermore, there is no clear recommendation regarding a minimum time interval between the delivery of CDK4/6 inhibitors and SABR. Nonetheless, when combining CDK4/6i with SABR, it was proposed that SABR should be performed without dose reduction and without using more SABR fractions compared with SABR without CDK4/6i.

Thus, gaining knowledge about the safe and effective application of such a combined treatment is important. Herein, we present a retrospective analysis of breast cancer patients treated at our institution with the CDK4/6i combined with SABR.

## Methods

### Study group and treatment

We performed a retrospective analysis of patients with ABC who received palbociclib, ribociclib, or abemaciclib and SABR in our institution between 2018 and March 2023. All patients were discussed at a multidisciplinary tumour board.

The majority of patients underwent systemic treatment in the Breast Unit, while radiation therapy procedures were carried out in the Radiotherapy Department except for 2 patients (one treated with Gammaknife and the second with Linear Accelerator). Thus, all data collected were derived from real-life settings, and no supplementary visits associated with the study were performed. The choice of CDK4/6i agent and SBRT was the treating physician’s decision. According to the prescribing information, the CDK4/6i dose was modified for adverse events (AE). In our centre, SABR was performed with CyberKnife (Accuray, Sunnyvale, CA, USA) or Linear Accelerator.

The study’s primary outcome was 2-year progression-free survival (PFS), whereas 2-year local control (LC) and 3-year OS were secondary outcomes.

Contrast-enhanced CT (CE-CT) served as the primary imaging modality in all patients. Baseline CE-CT was performed, followed by subsequent scans every 3 months to assess treatment response.

Additionally, PET-computed tomography with 2-deoxy-2-[fluorine-18]fluoro-d-glucose (PET/CT) was performed in a subset of patients at baseline and during CDK4/6i treatment.

Tumour response was evaluated according to Response Evaluation Criteria in Solid Tumours version 1.1 criteria (RECIST v. 1.1) and determined as a complete response (CR), partial response (PR), progressive disease (PD), or stable disease (SD).^13^

**Figure 1.**
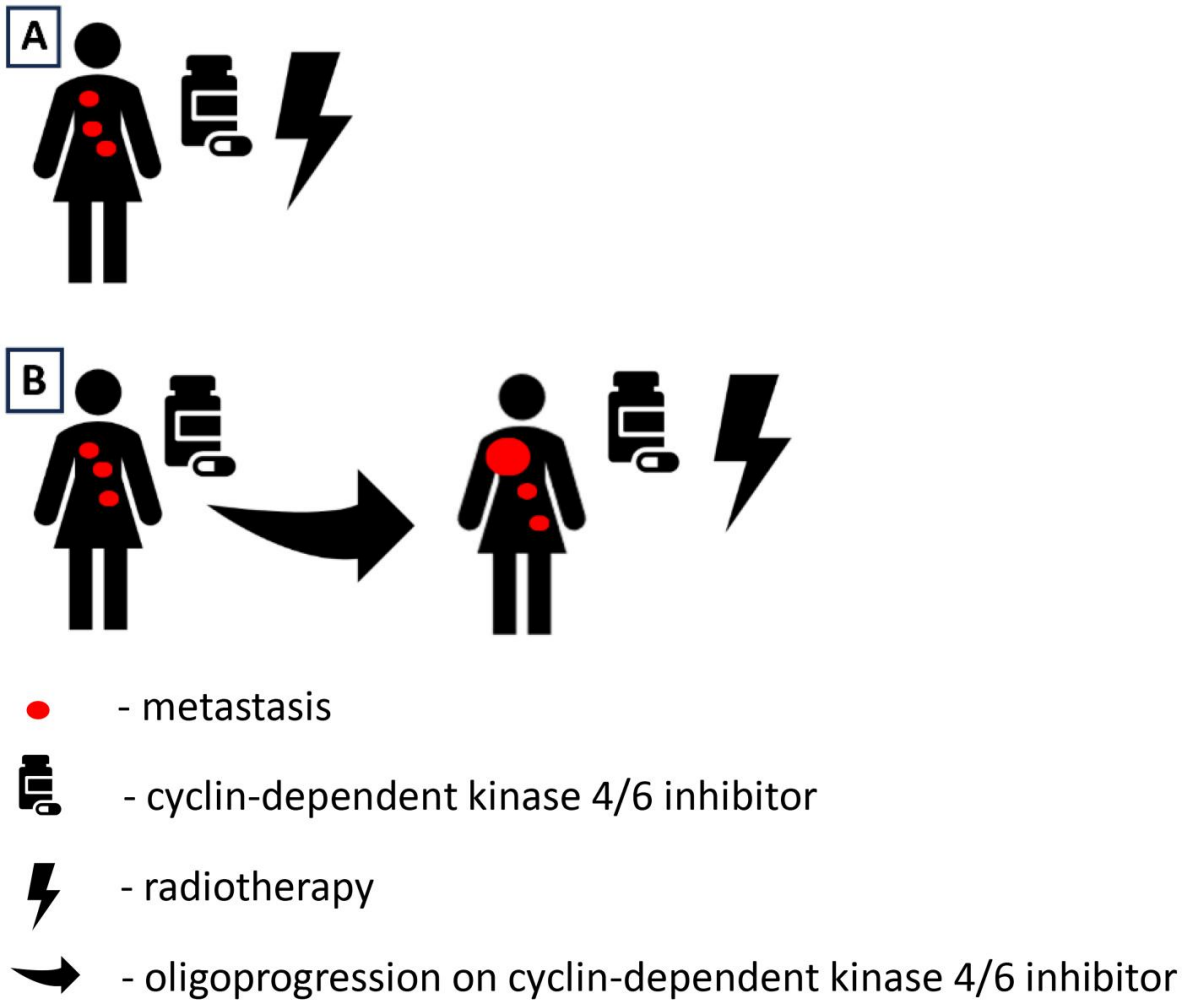
Oligometastatic (A) and oligoprogressive (B) disease; the relation between cyclin-dependent kinase 4/6 inhibitor and stereotactic ablative radiotherapy timing.

Oligometastatic patients was defined as up to 5 metastases in a maximum of 3 organs. Oligoprogression (OPD) was defined as a PD of a small number of lesions on imaging without fulfilling the criteria of PD. Oligometastatic patients and OPD states are depicted in Figure 1.

Overall survival was measured from the date of diagnosis of the metastatic disease to the time of death or last follow-up. Progression-free survival was defined as the time from the start of CDK4/6i treatment to the occurrence of PD or death. Patients who died before disease progression were considered censored at the time of death. Local control was defined as the time from SBRT completion to tumour progression of the irradiated site.

### Statistical analysis

Categorical variables were shown as frequencies and percentages. Continuous data were summarized as median values with interquartile ranges (IQR, 25%-75%). PFS, OS, and LC were estimated using the Kaplan-Meier method, and 95% CIs for the survival curves were calculated. Survival,^14^ survminer,^15^ and survMisc^16^ packages were used. All computational analysis were performed in the R environment for statistical computing version 4.0.1 “See Things Now” released on 6 June 2020 (R Foundation for Statistical Computing, Vienna, Austria, http://www.r-project.org, accessed on 20 February 2023).

All procedures performed in the study were in accordance with the 1964 Helsinki Declaration with later amendments and with the ethical standards of the institutional Ethics Committee, which approved the study (no. KB/430-27/22).

## Results

### Patient and treatment characteristics

**Figure 2.**
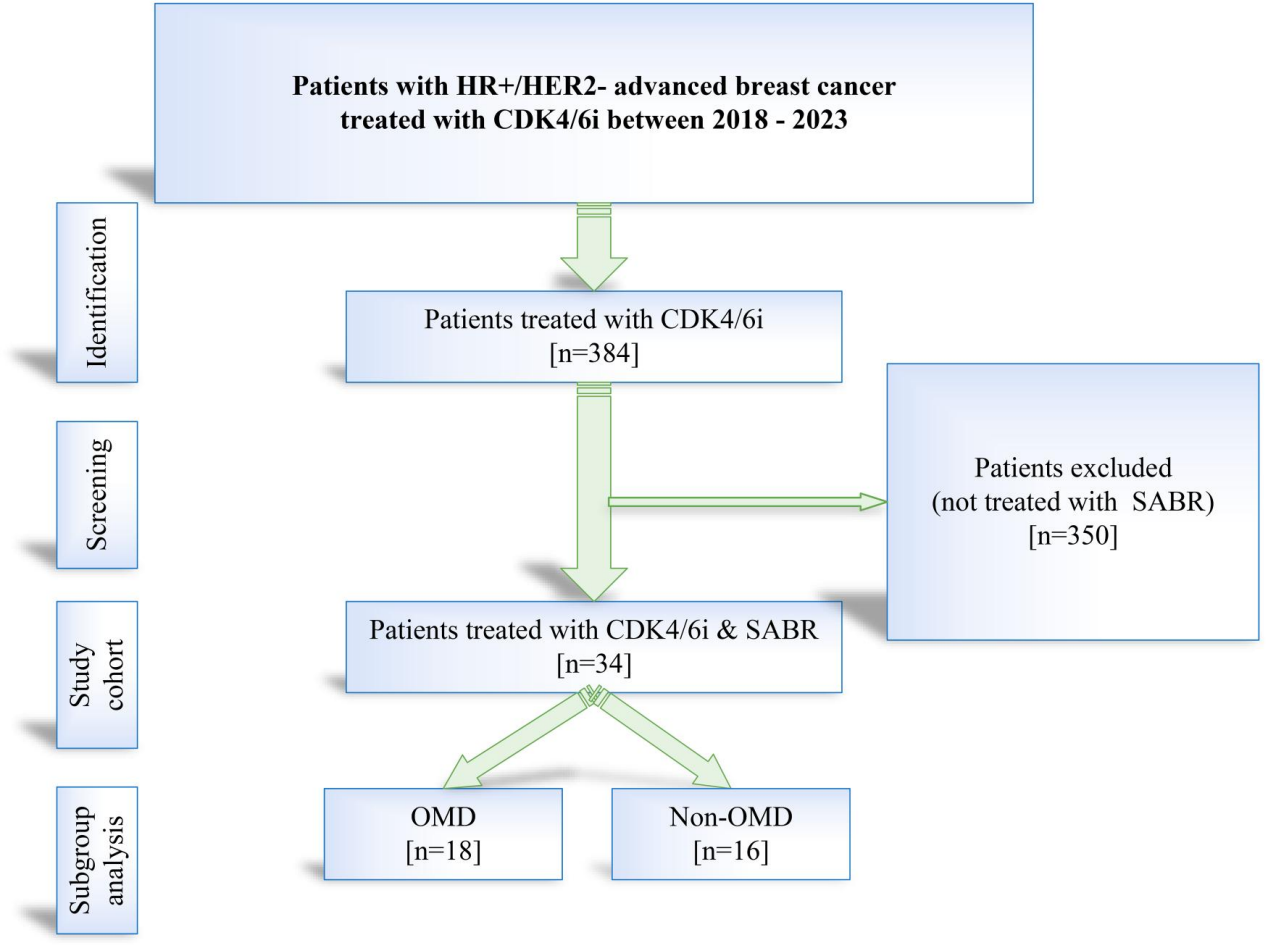
Consort diagram describing the population of patients included in the study. Abbreviations: CDK4/6i = cyclin-dependent kinase 4/6 inhibitors; *n* = number; non-OMD = non-oligometastatic disease; OMD = oligometastatic disease; SABR = stereotactic ablative radiotherapy.

In our institution, 384 patients were treated with CDK4/6i. Among them, 34 patients received stereotactic RT. The study participants are depicted in Figure 2. The median age was 59 years, and 10 patients were younger than 50 years. Fifteen patients were diagnosed with de novo disease, whereas 19 patients had recurrent disease. Twenty-seven patients were treated in the first line and 7 in the second. Nineteen patients had bone-only disease. Twenty-three patients received chemotherapy previously, including 8 patients with chemotherapy treatment within 1 year before CDK4/6i commencement. Twenty-two patients were treated with ribociclib, 7 with palbociclib, and 5 with abemaciclib. Letrozole as an endocrine compound was implemented in 24 patients, whereas the remaining 10 received fulvestrant.

Forty-four stereotactic treatments were performed. In the OMD setting, most patients received RT before CDK4/6i implementation or shortly after the beginning of CDK4/6i treatment. In OPD, RT was usually implemented during CDK4/6i treatment after variable courses of CDK4/6i cycles. Three patients were irradiated due to the oligoprogressive disease: liver metastasis, lung metastasis, or primary breast tumour. Twenty-three SBRT were performed concomitantly with the CDK4/6i treatment. Twenty-one SBRT were delivered before the CDK4/6i commencement with a median interval of 41 days (IQR 18.5-75 days). The most prolonged delay between SRS and CDK4/6i treatment was in a patient with isolated brain metastasis and poor performance status, which equals 107 days. Bones were the most common treatment site (70.5%), including 20 treatments in the spine. Radiation therapy details are shown in Tables 1 and 2. Radiation sites in patients who received cyclin-dependent kinase 4/6 inhibitors concurrently vs sequentially are depicted in Table S1.

**Table 1.** Radiation sites.

Location	No. of Tx *n* = 44 (%)
Cervical spine	7 (15.9)
Thoracic spine	6 (13.6)
Lumbar spine	7 (15.9)
Pelvis	6 (13.6)
Other bones (clivus, sphenoid bone, extremities, ribs)	5 (11.4)
Brain	6 (13.6)
Lung	3 (6.8)
Liver	2 (4.5)
Other (breast, retroperitoneum)	2 (4.5)

Abbreviation: Tx = treatment.

**Table 2.** Radiation technique and dose (Gy).

Technique	Total dose	Dose per fraction	No. of Tx *n* = 44 (%)
CyberKnife	36	12	3 (6.8)
	27	9	1 (2.3)
	26	5.2	1 (2.3)
	24	8	1 (2.3)
	18	6	3 (6.8)
	15	15	5 (11.4)
	16	8	1 (2.3)
	12	6	1 (2.3)
Linac stereotactic radiation therapy	54	18	2 (4.5)
	45	15	4 (9.1)
	36	12	5 (11.4)
	30	10	6 (13.6)
	25	5	4 (9.1)
	24	12	1 (2.3)
	24	8	4 (9.1)
	22.5	4.5	1 (2.3)
GammaKnife	20	20	1 (2.3)

Abbreviation: Tx = treatment.

### Treatment efficacy

Contrast-enhanced CT was performed in all patients at baseline and every 3 months thereafter. Twenty-one patients also had baseline PET/CT, including the majority of patients with metastases limited to the skeletal system (14 patients out of 19). Additionally, in 9 patients, PET/CT was performed during CDK4/6i treatment. However, those studies were performed at various time points. According to RECIST 1.1, 1 patient had CR, 5 PR, 24 SD, and 4 had disease progression as the best response. Four patients achieved a complete metabolic response in PET/CT.

At the time of data cut-off, 28 patients were still alive (82.4%), 21 patients were still treated with CDK4/6i, and 2 patients had to discontinue CDK4/6i due to toxicity with SD on endocrine monotherapy continuation. Eleven patients discontinued CDK4/6i due to disease progression. The leading site of disease progression was the liver (outside irradiated site, 63.6%, 7 patients out of 11 patients who progressed). The other progression sites included bones, breast tumour, and central nervous system in 2 patients.

**Figure 3.**
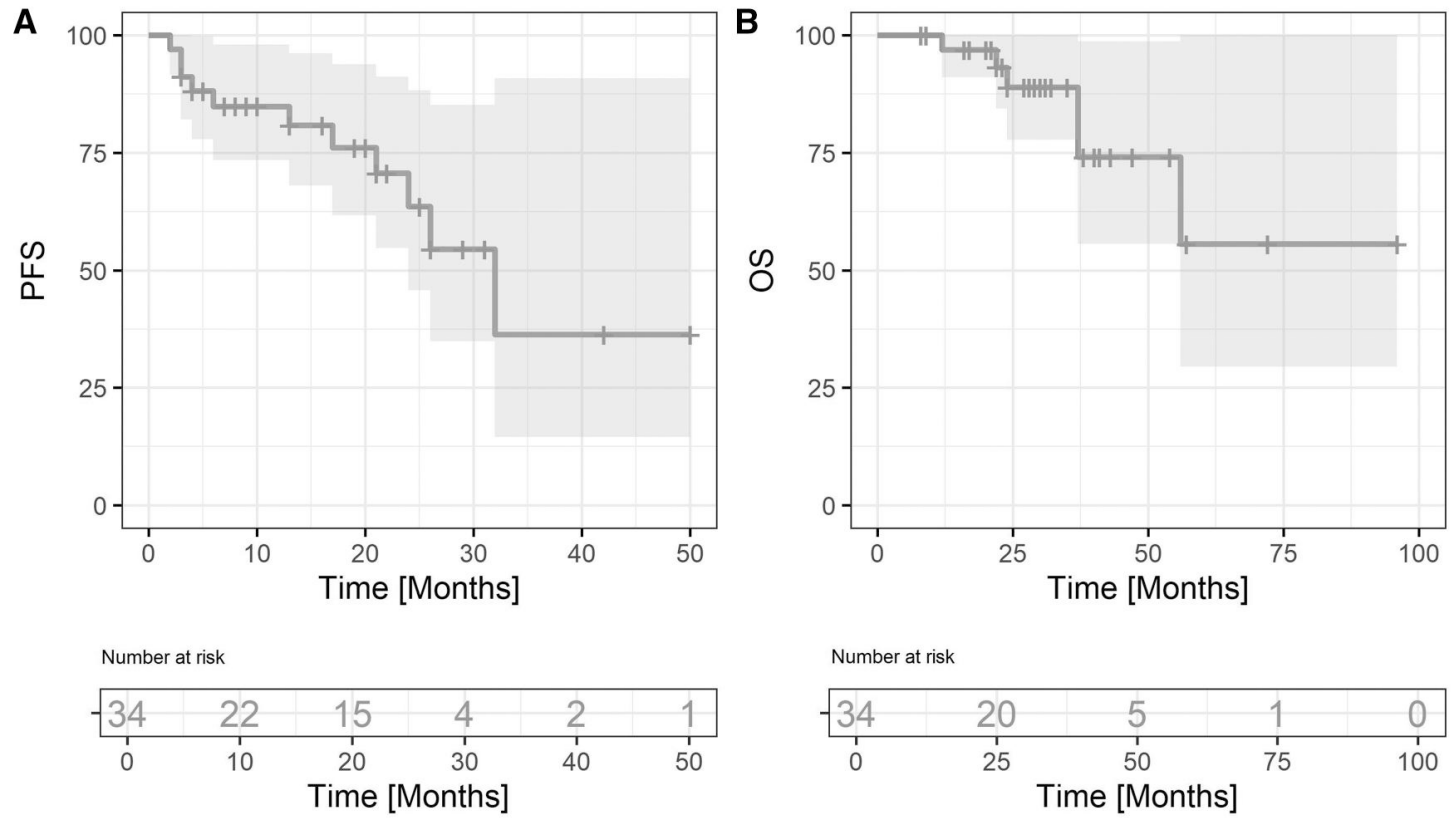
Treatment efficacy. (A) Progression-free survival (PFS) and (B) overall survival (OS).

Two-year PFS was 63.6% (95% CI, 45.8-88.3), and the median PFS was 32 months. Three-year OS was 88.9% (95% CI, 77.7-100). Two-year local control was 92.7% (95% CI, 83.4-100). Only 2 patients progressed in the irradiated site, both in brain metastases. Median OS and LC were not reached. PFS and OS are shown in Figure 3.

In 3 patients irradiated for oligoprogressive sites SABR was effective; however, only 1 patient had SD at the time of data cut-off. The remaining 2 discontinued treatments due to disease progression outside irradiated sites.

### Safety analysis

The most common AEs were associated with CDK4/6i, with neutropenia leading. Almost half of the patients had CDK4/6i dose reduction (44%), mainly due to neutropenia (14 out of 15 patients with dose reduction). Within 2 weeks after completion of SABR, given concomitantly with CDK4/6i, 2 episodes of grade 3 neutropenia, and 1 grade 4 neutropenia were reported. In the first patient, SABR for 2 lumbar vertebrae (L3-4, PTV 103.8 cm^3^, 18 Gy in 3 fractions) and ischial bone (PTV 13.5 cm^3^) was performed during the first CDK4/6i cycle. Similar episodes of neutropenia were observed in the remaining 2 patients before SABR. Only 1 dose reduction was not haematologic-related. In this case, the patient treated with abemaciclib developed grade 3 diarrhoea. This patient received SABR for brain metastasis.

Two patients discontinue CDK4/6i due to toxicity for the following reasons. One patient received SBRT 45 Gy delivered in 3 fractions to the right and left lung metastases 8 weeks before CDK4/6i commencement and was diagnosed with bilateral pulmonary fibrosis 8 months after RT completion. Fibrosis was observed in large areas of both lungs, even outside the low-dose area. Dyspnoea G2 resolved after treatment with steroids. Radiation-induced pneumonitis was suspected; however, pulmonologists considered previous COVID-19 infections a differential cause. The second patient started CDK4/6i and received 25 Gy in 5 fractions to the fifth lumbar vertebra. After completing RT, the patient was admitted to the hospital due to vomiting resulting in worsening performance status and electrolyte imbalance. Both patients recovered to their previous performance status with SD on letrozole continuation. Brain radiation was well tolerated except for 2 patients with prolonged fatigue; 1 patient presented with ECOG 2 and hemiparesis before RT, and the second had G3 fatigue for 10 days, which resolved after that. Both received RT before CDK4/6i commencement (time interval 107 and 35 days, respectively). One patient was hospitalized due to atrial fibrillation 1 month after completing SRS to the clivus; thus, it was considered unrelated to RT. No difference in high-grade toxicity was observed between the sequential and concurrent SABR. All SBRT treatments were performed as planned without treatment interruptions or discontinuations. No G5 toxicity was found. With a median follow-up of 22.1 months (IQR 9.2-28.4 months), no other radiation-related high-grade toxicity was observed. No cases of symptomatic radiation necrosis in our study were observed, neither in patients undergoing brain SABR nor in the non-SABR cohort.

### Cyclin-dependent kinase 4/6 inhibitors dose reduction rates compared to the non-SABR population

Among 384 patients, 34 patients received SABR. Out of the remaining 350 patients who did not undergo SBRT, 99 received palliative conventional RT. Among all patients treated with CDK4/6i (384 patients), 204 required CDK4/6i dose reduction (53.1%). Of patients who received palliative conventional RT, 49.5% required CDK4/6i dose reduction (49 out of 99). Among patients treated with SABR, 44.1% required CDK4/6i dose reduction (15 out of 34). There was no significant difference in CDK4/6i dose reduction between patients who received SABR compared to all patients who did not receive SABR (*P* = .29). Additionally, no significant difference was observed in CDK4/6i dose reduction when comparing patients treated with SABR to those who received palliative conventional RT (*P* = .69).

### Subgroup analysis

Since our group consisted of heterogeneous, non-selected patients, we performed a subgroup analysis. Nineteen patients had limited metastasis (≤5). Among patients with brain metastases, 5 had multiple metastases outside the brain. One patient had isolated brain metastasis; however, we included this patient in the non-OMD group since these patients were excluded from most randomized trials with OMD SBRT. Furthermore, CDK4/6i treatment was postponed due to poor performance status and started after improving to ECOG 2, and declined soon after. Thus, 18 patients were included in the OMD subgroup and 16 in the non-OMD subgroup. A comparison between these 2 subgroups is shown in Table 3.

**Table 3.** A comparison of patients in the OMD and non-OMD subgroups.

Characteristics	OMD (*n* = 18)	Non-OMD (*n* = 16)
Age (median)	66	51
Age under 50 years	3	7
De novo metastatic	12	3
**Recurrent disease**		
1st line of Tx	16	11
2nd line of Tx	2	5
Bone-only disease	13	6
Bone + visceral mets	4	9
Visceral mets without bone	1	1
Previous CHT	11	12
Previous CHT within 1 year	7	1
ECOG 0	8	0
ECOG 1	9	7
ECOG 2	1	9
**CDK4/6i**		
Ribociclib	12	10
Palbociclib	3	4
Abemaciclib	3	2
**Endocrine Tx**		
Aromatase inhibitor	15	9
Fulvestrant	3	7
CDK4/6i dose reduction	9	6

Abbreviations: CHT = chemotherapy; CDK4/6i = cyclin-dependent kinase 4/6 inhibitors; ECOG = Eastern Cooperative Oncology Group performance status; mets =metastases; non-OMD = non-oligometastatic disease; OMD = oligometastatic disease; Tx = treatment.

### Treatment efficacy comparison between the OMD subgroup and non-OMD patients

**Figure 4.**
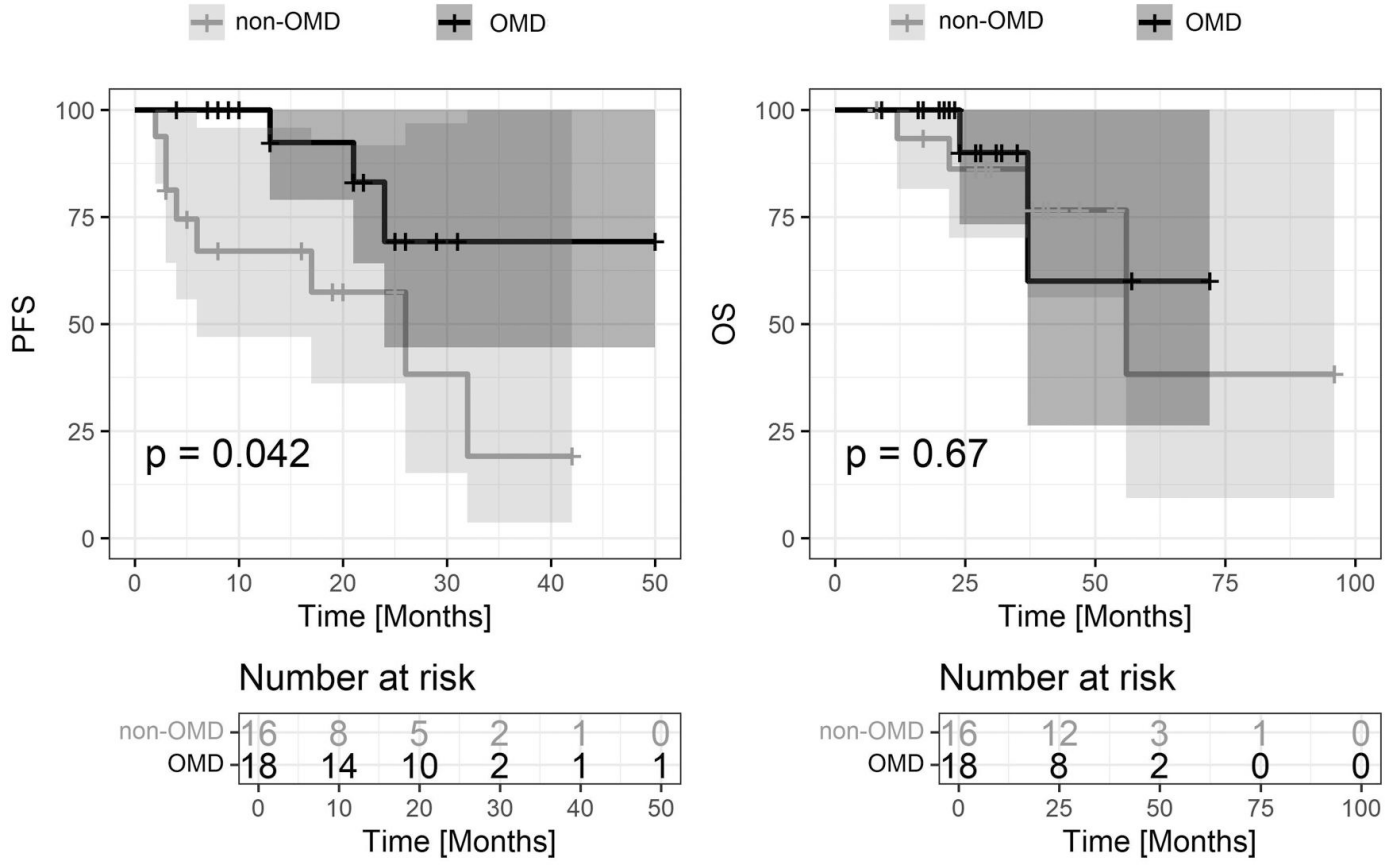
Comparison of progression-free survival (PFS, A) and overall survival (OS, B) in the group of oligometastatic disease (OMD, black line) and non-oligometastatic disease (non-OMD, grey line).

A significant difference was found at 2-year PFS, which was 69.2% (95% CI, 44.5-100) in OMD compared with 57.4% (95% CI, 36-91.7) in the non-OMD subgroup (*P* = .042). Three-year OS was 90% (95% CI, 73.2-100) in the OMD compared with 86.2% (95% CI, 70-100) in the non-OMD subgroup (*P* = .67). Median PFS and OS were not reached in the OMD subgroup, whereas it was 26 and 56 months in the non-OMD group, respectively. The results are shown in Figure 4.

## Discussion

CDK4/6i changed the landscape of the management of HR+/HER2− ABC patients and represent the current standard of treatment in first- and second-line settings. Despite published results of prospective trials concerning the SABR in metastatic breast cancer patients, data regarding the combination with CDK4/6i are lacking. Furthermore, recent EORTC-ESTRO recommendations reported no consensus on whether or not CDK4/6i can be administered on the same days as SBRT delivery and the minimum time interval between delivery of CDK4/6 inhibitors and SBRT.^12^ Two meta-analyses of CDK4/6i combined with radiation therapy were recently published^17^^,^^18^; however, the patients treated with SBRT were underrepresented. Thus, we present a retrospective analysis of breast cancer patients treated at our institution with the CDK4/6i combined with SABR.

A common approach in metastasis-directed stereotactic RT clinical trials was to include patients with multiple cancer types.^19^ SABR appeared to be relatively safe, with clinically acceptable rates of early and late G3-G5 toxic effects <13%. One-year local control, 1-year OS, and 1-year PFS were 94.7%, 85.4%, and 51.4%, respectively, and breast cancer patients comprised 13.1%.^20^ In the SABR-5 study, patients with genuine oligometastatic, oligoprogressive, and induced oligometastatic disease were enrolled, including 11% with breast cancer, and the median PFS was 15 months, whereas the 3-year LC was 87%.^21^ Chmura et al conducted a phase II trial to determine the efficacy of metastases-directed treatment with SBRT or SR in addition to standard-of-care systemic therapy in breast cancer patients.^22^ Sixty percent had 1 metastasis, and most patients were HR+/HER2−. With the median follow-up of 30 months, the median PFS was 19.5 months, 2-year and 3-year PFS were 46.8 and 38.1, respectively, whereas the 3-year OS was 68.9%. In our study, survival results concerning oligometastatic breast cancer patients are better, with a 3-year OS of 90%, a 2-year PFS of 69.2%, and a 2-year LC of 92.7%. Moreover, the 2-year LC in the non-brain metastases was 100%. Nonetheless, the study of Chmura et al was activated before the advent of CDK4/6i.^22^

Patients with HR+/HER2− ABC, especially oligometastatic disease, have a relatively long OS and several therapy options; thus, the effect of both therapy and the disease on the patient’s symptoms and functioning is essential.^23^ CDK4/6i did not worsen health-related quality of life when compared with endocrine alone.^24^ Since the retrospective character of our study, we did not evaluate health-related quality of life parameters. Such outcomes need to be addressed in prospective trials. Nonetheless, we did not observe enhanced toxicity with SBRT addition or worsening CDK4/6i treatment tolerance. A median follow-up of 22 months is comparable to other trials, such as 25 and 30 months in the study of Palma et al and Chmura et al, respectively.^19^,^22^

SABR, as a metastases-directed treatment of a few progressive lesions, may allow continuing the same line of systemic therapy. Recently, the results of AVATAR, a multicentre phase II trial of stereotactic RT with CDK4/6i treatment in ABC, were presented.^25^ Thirty-two patients with oligoprogressive disease were recruited, showing a higher than anticipated median time to change in systemic treatment. In our study, SABR was effective in local control; nonetheless, the primary issue was further progression outside the radiation field.

At present, we anticipate the results of prospective phase II studies regarding a combination of CDK4/6i treatment and RT in specific breast cancer patients populations: CLEAR trial (NCT03750396), ASPIRE trial (NCT03691493), and PALATINE trial (NCT03870919).

The optimal timing of SABR remains unknown. Progressive disease as the best overall response was reported in 5.7% of patients in first-line ribociclib treatment.^26^ Four patients in our study (11.8%) progressed at the first radiologic assessment after 3 months of CDK4/6i treatment. Whether these patients, defined as early-progressors, benefit from SABR requires further validation. In the SABR-COMET phase II study,^27^ patients with a controlled primary tumour for at least 3 months were eligible.

Our studied population, comprising 34 patients, is quite large. In the SABR-COMET, 18 patients were diagnosed with breast cancer, and 13 received SABR.^19^ In the study of Chmura et al,^22^ 60 patients received SBRT/RT in addition to the systemic treatment. One of the strengths of our study was that all patients received homogenous treatment with CDK4/6i, whereas systemic therapy was highly heterogeneous in other studies. Furthermore, older trials applied chemotherapy as a systemic backbone in the multimodality treatment of OMD in breast cancer patients.

Optimal systemic therapy is crucial for managing metastatic breast cancer.^9^ Recent advancements in treatments have notably improved survival in this population. Nonetheless, the treatment choice highly depends on the intrinsic subtype of the breast cancer. For instance, triple-negative breast cancer may be treated with drugs like pembrolizumab^28^ and sacituzumab govitecan,^29^ while HER2-positive breast cancer exhibited high response rates to trastuzumab deruxtecan^30^ and tucatinib.^31^ Similarly, olaparib^32^ and talazoparib^33^ are used for patients with a germline BRCA (BReast CAncer gene) mutation, while alpelisib is effective in PIK3CA-mutated tumours.^34^ It is important to note that the outcomes and side effects of adding SABR can vary widely among these different treatment scenarios.

Our results should be considered in the context of the limitations of this study. Current evidence does not support the ablative approach of metastatic sites as a standard approach; thus, the allocation to SABR was driven by clinical consideration, not randomization, resulting in baseline confounding bias. Contrary to other SABR trials in OMD,^19^,^22^ most of our study population was not treated to all metastatic sites. Among the OMD group, 11 patients were irradiated to all metastatic sites. However, due to the low number of patients, it is too early to conclude. Furthermore, the subgroup analysis may be biased, particularly as there was a higher representation of patients with bone-only disease within the OMD group. This imbalance could potentially influence the more prolonged survival compared to the non-OMD group.

Data regarding the effectiveness of PET/CT in monitoring patients with ABC during CDK4/6i are limited.^35–37^ Among the patients studied, 4 exhibited a complete metabolic response. Nevertheless, PET/CT scans were conducted in just 9 patients during their CDK4/6 inhibitor treatment. Moreover, these scans were performed at various stages of treatment. Consequently, it is challenging to determine the reliability of PET/CT scans for evaluating responses in patients undergoing SABR and CDK4/6 inhibitor treatment due to the inconsistency in timing.

Both CDK4/6i and SABR may be involved in an immune response. Results from the RIBECCA trial showed that treatment with ribociclib significantly affects the peripheral innate and adaptive immune response in HR+ breast cancer patients.^38^ Ribociclib was used for treatment in most patients in our study. SABR may boost host antitumour immune responses by inducing locally and systemically immunomodulating effects in oligometastatic breast cancer patients.^39^ In that study, treatment with 3 daily doses of 10 Gy was implemented, and this was also our study's most commonly utilized schedule. Whether a combination of CDK4/6i and SABR enhances immune response in synergistic mechanisms of action requires further validation.

## Conclusion

Overall survival and PFS of ABC patients treated with stereotactic ablative radiation therapy added to cyclin-dependent kinase 4/6 inhibitors is relatively high in oligometastatic patients. The addition of SABR to CDK4/6i seems to be safe and effective. Whether it is a bias of patient selection or an actual synergistic effect requires further validation in prospective studies.

## Supplementary Material

tqae138_Supplementary_Data
