# Supplementary material for: Cyclin-dependent kinase 4/6 inhibitors combined with stereotactic ablative radiotherapy in oligometastatic HR-positive/HER2-negative breast cancer patients
Source: Br J Radiol. 2024 Aug 9;97(1162):1627–35. doi: 10.1093/bjr/tqae138 (PMC11417346; doi:10.1093/bjr/tqae138)
Supplement: tqae138_Supplementary_Data [file tqae138_supplementary_data.docx]

**Table 1 Suppl**. Radiation sites in patients who received cyclin-dependent kinase 4/6 inhibitors concurrently vs. sequentially.

| SABR site | SABR conc  [No of Tx: n=23] | SABR seq.  [No of Tx: n=21] |
| --- | --- | --- |
| Cervical spine | 2 (8.7%) | 5 (23.8%) |
| Thoracic spine | 4 (17.4%) | 2 (9.5%) |
| Lumbar spine | 4 (17.4%) | 3 (14.3%) |
| Pelvis | 3 (13.0%) | 3 (14.3%) |
| Other bones  (clivus, sphenoid bone, extremities, ribs) | 4 (17.4%) | 1 (4.8%) |
| Brain | 1 (4.35%) | 5 (23.8%) |
| Lung | 1 (4.35%) | 2 (9.5%) |
| Liver | 2 (8.7%) | 0 (0%) |
| Other (breast, retroperitoneum) | 2 (8.7%) | 0 (0%) |

Abbreviations: SABR – stereotactic ablative radiotherapy; conc – concurrent; seq – sequential; No of Tx – number of treatments
